# Exercise, Nrf2 and Antioxidant Signaling in Cardiac Aging

**DOI:** 10.3389/fphys.2016.00241

**Published:** 2016-06-17

**Authors:** Madhusudhanan Narasimhan, Namakkal S. Rajasekaran

**Affiliations:** ^1^Department of Pharmacology and Neuroscience, Texas Tech University Health Sciences CenterLubbock, TX, USA; ^2^Cardiac Aging and Redox Signaling Laboratory, Center for Free Radical Biology, Division of Molecular and Cellular Pathology, Department of Pathology, University of Alabama at BirminghamBirmingham, AL, USA; ^3^Division of Cardiovascular Medicine, Department of Medicine, University of Utah School of MedicineSalt Lake City, UT, USA; ^4^Department of Exercise Physiology, College of Health, University of Utah School of MedicineSalt Lake City, UT, USA

**Keywords:** oxidative stress, Nrf2, exercise, antioxidant signaling, cardiac aging

## Abstract

Aging is represented by a progressive decline in cellular functions. The age-related deformities in cardiac behaviors are the loss of cardiac myocytes through apoptosis or programmed cell death. Oxidative stress (OS) and its deleterious consequence contribute to age-related mechanical remodeling, reduced regenerative capacity, and apoptosis in cardiac tissue. The pathogenesis of OS in the elderly can predispose the heart to other cardiac complications such as atherosclerosis, hypertension, ischemic heart disease, cardiac myopathy, and so on. At the molecular level, oxidant-induced activation of Nrf2 (Nuclear erythroid-2-p45-related factor-2), a transcription factor, regulates several genes containing AREs (Antioxidant Response Element) and bring the respective translates to counteract the reactive radicals and establish homeostasis. Myriad of Nrf2 gene knockout studies in various organs such as lung, liver, kidney, brain, etc. have shown that dysregulation of Nrf2 severely affects the oxidant/ROS sensitivity and predispose the system to several pathological changes with aberrant cellular lesions. On the other hand, its gain of function chemical interventions exhibited oxidant stress resistance and cytoprotection. However, thus far, only a few investigations have shown the potential role of Nrf2 and its non-pharmacological induction in cardiac aging. Therefore, here we review the involvement of Nrf2 signaling along with its responses and ramifications on the cascade of OS under acute exercise stress (AES), moderate exercise training (MET), and endurance exercise stress (EES) conditions in the aging heart.

## Background

Redox homeostasis is a delicate steadiness between oxidants and antioxidants in a setting of intracellular milieu. In general, human cells constantly undergo numerous chemical reactions and modifications every second requiring the constant need for oxygen and other compounds for a plethora of processes. These obligatory processes involving oxygen could produce highly reactive oxygen species within the cell which when uncontrolled leads to oxidative stress (OS) (Uttara et al., 2009). OS can be potentially dangerous to numerous physiological functions ranging from gene expression to cell survival/apoptosis and interrupt the redox control within the cell (Harman, 1981; Capell et al., 2007; Zhou et al., 2014). Disturbance of redox homeostasis and subsequent “disruption of redox signaling and regulation” plays a critical role in patho-mechanism of several human diseases including major cardiovascular problems, which when left untreated may prove fatal (Burgoyne et al., 2012). The heart being an aerobic organ consumes ~8–15 ml O_2_/min/100 g tissue at resting state that can be increased to >70 ml O2/min/100 g myocardial tissue during vigorous exercise (West, 1981; Braunwald, 2001). Thus, the role of oxygen and oxygen-related processes are major determinants of its vitality wherein, a moderate tilt in the redox state of the cell may tip toward the increased production of oxidants causing oxidative damage leading to cardiac dysfunction and heart failure (Capell et al., 2007; Zhou et al., 2014).

Normally, the cellular system deal with the excessive oxidative burden through activation of several signaling proteins and transcription factors such as NFκB, AP1, HIF1α, P53, and Nrf2 [nuclear factor (erythroid-derived 2)-like 2] that transcriptionally controls antioxidant signaling (Haddad et al., 2000; Muthusamy et al., 2012). However, the amplitude of response may vary widely depending on the severity of oxidant/antioxidant imbalance, inherent tolerance, genetic makeup, environmental context and several other regulatory factors resulting in either adaptation, protection, stress and/or destruction (Rahal et al., 2014).

**Figure 1:**
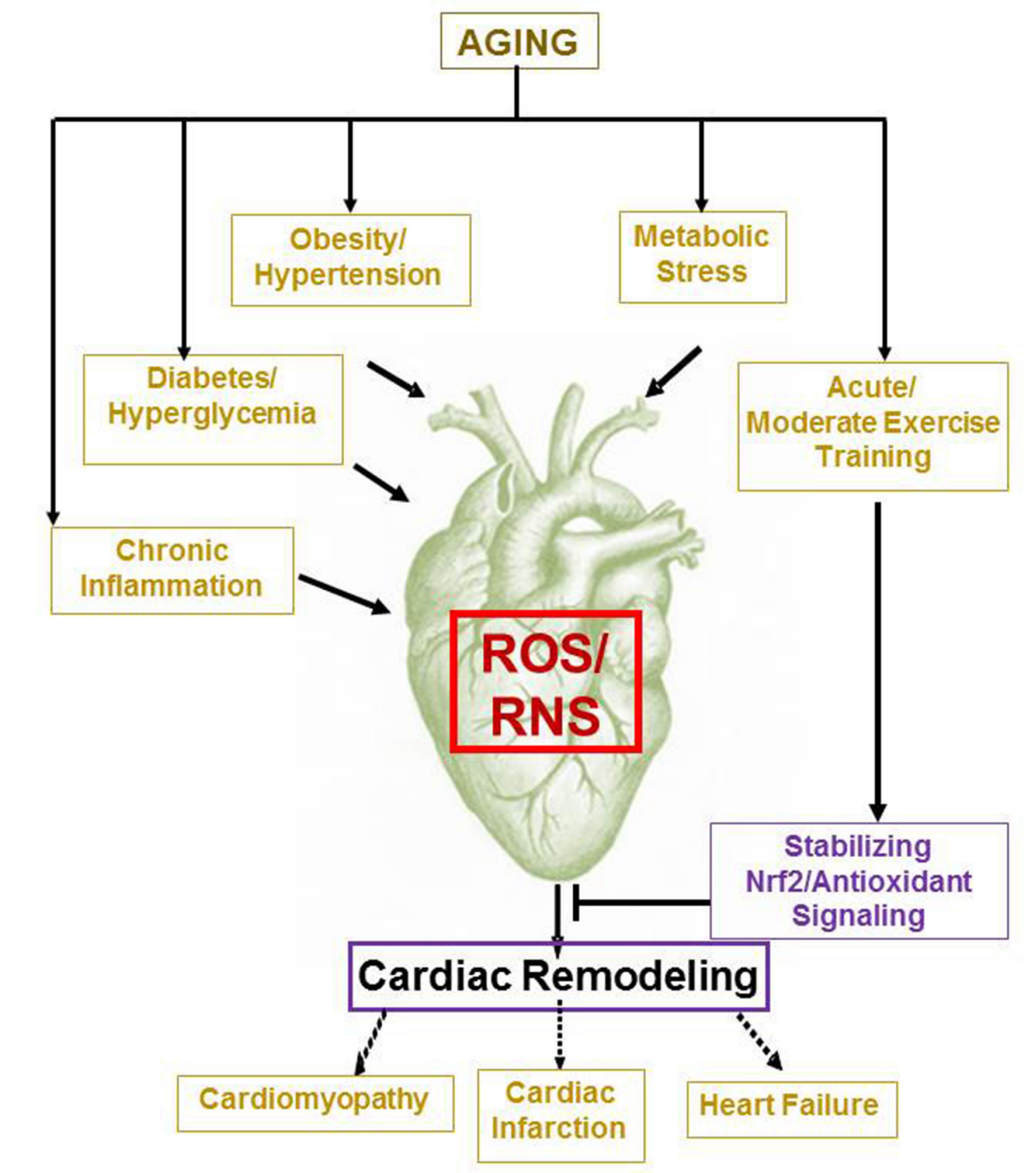
**Aging and age associated pathological processes induce cardiac remodeling**. While metabolic and chronic conditions such as obesity, diabetes, hypertension, inflammation, etc. promotes oxidative stress dependent myocardial remodeling and dysfunction, moderate exercise training is likely to improve cardiac health by stabilizing Nrf2/Antioxidant signaling on aging.

Relevant to these facts, uncontrolled OS has been identified as a potent constituent in the etiology of stroke, coronary heart disease, ischemia/reperfusion injury, atherosclerosis, and hypertension (Harman, 1981; Lakatta, 1994; Kajstura et al., 1996; Higami and Shimokawa, 2000). Importantly, recent epidemiological studies report an increased incidence of cardiovascular disease (CVD) among aged population (>65 years) in the United States in the past years (Karavidas et al., 2010; Roger et al., 2012; Wu et al., 2014; Mozaffarian et al., 2015). In 2012, the CDC released data that Coronary Heart Disease had a strong correlation with age and was the number one cause of mortality in the United States (Lakatta, 1994; Jennings et al., 1997). As an individual's age increases, the normal adaptations and modifications that were once occurring in the human body turns functionally defective on one hand (Figure 1) and on the other hand, the heart's ability to keep up the pressure and volume changes of the vascular system deteriorates leading to poor cardiac performance and myocardial decline (Gounder et al., 2012). Notably, age-associated OS has been identified as an independent factor to severely influence CVD and heart failure (Jennings et al., 1997; Capell et al., 2007; Collins et al., 2009; Gounder et al., 2012).

In 2013, the American Heart Association estimated 83.6 million Americans to have been diagnosed with at least one CVD (Mozaffarian et al., 2015). Among that population, 42.2 million are ~60 years of age or older, with the mortality rate of those above the age of 75 being 66% (Mozaffarian et al., 2015). Though much effort has gone into understanding the cardiovascular aging and aging-cardiovascular pathway, a sharp translational divide still exists between the basic mechanistic elucidations and clinical investigations and/or approaches.

It is widely agreed that aging uncouples the electron transport and ATP production resulting in free radical elaboration and acceleration of OS. Concurrently, with the age-related decline in the production and activity of biological antioxidants, there is an overburden of ROS/RNS (Zhou et al., 2014). A component of the cellular antioxidant defense mechanism is the multitude of cytoprotective genes bearing the antioxidant response element (AREs) such as NAD(P)H-quinone oxidase-1 (NQO1), heme oxygenase (HO1), Υ-glutamyl cysteine ligase-catalytic (GCLC), Υ-glutamyl ligase-modulatory (GCLM), glucose-6-phosphate dehydrogenase (G6PD), glutathione peroxidase-1 (GPX1), glutathione peroxidase-2 (GPX2), glutathione reductase (GSR), and catalase to scavenge the radicals (Osburn and Kensler, 2008; Muthusamy et al., 2012), which are regulated by nuclear factor erythroid-2-p45-related factor-2 (Nrf2), a transcription factor belonging to Cap “n” Collar (CNC) family of basic leucine zipper (bZip). Although Nrf2 is originally discovered as a transcription factor that can bind to erythroid transcription factor 2 (NF-E2) binding motif, due to its similarity to antioxidant response element (ARE) binding motif (Andrews et al., 1993), later it has been established as a major regulator of several genes containing the ARE sequence. Notably, Nrf2 is shown to possess an evolutionarily conserved role in protection against OS (Mukaigasa et al., 2012). In addition, mild to moderate level of ROS has been shown to activate the Nrf2 activation and up-regulate antioxidants and trigger detoxification mechanisms in the vasculature of young animals as an adaptive response (Warabi et al., 2007; Wu et al., 2014). On the other hand, a prolonged oxidative condition in a 5-month chronic hyperglycemia model severely downregulated Nrf2 and blunted its antioxidant response in the heart of mice as opposed to 2-month old hyperglycemic model (Tan et al., 2011). Notably, the autopsied heart specimens from diabetic patients displayed decreased nuclear Nrf2 levels accompanied by increased 3-nitrotyrosine compared to control hearts (Tan et al., 2011) indicating that Nrf2 can be bi-modally regulated depending on the ROS concentration in the heart.

**Figure 2:**
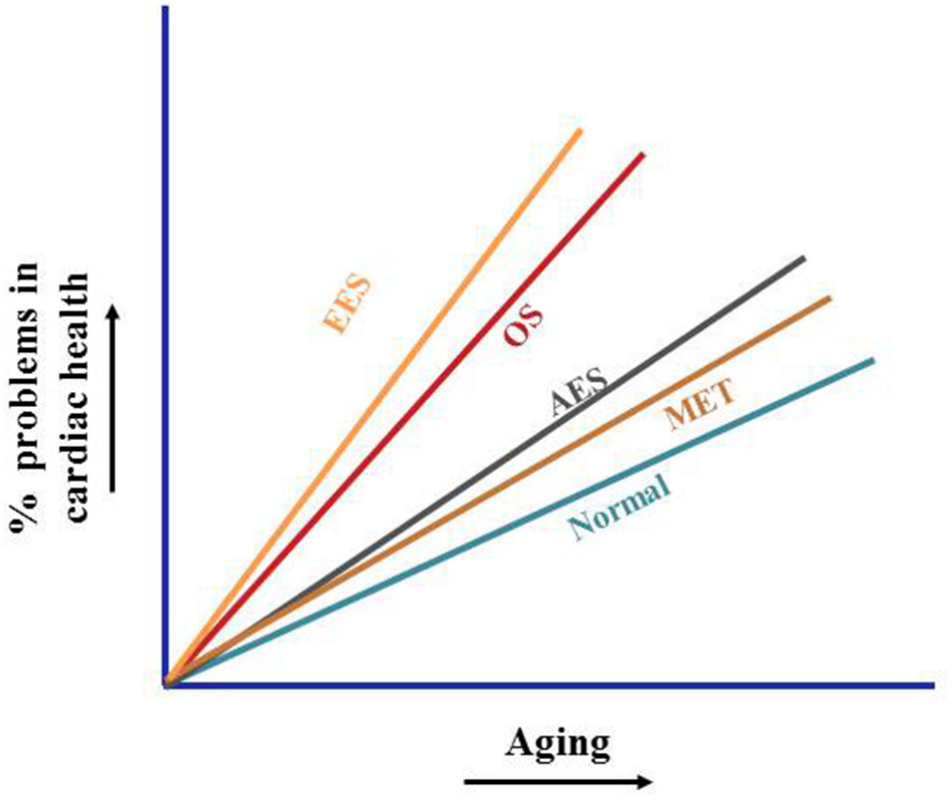
**Non-pharmacological activation of cardiac defense**. Age-dependent decline of Nrf2 result in chroic oxidative stress and subsequent induction of pathological processes accelerating cardiac complication. Acute exercise promotes transient transcriptional induction of cytoprotective genes, endurances exercise develops severe oxidative stress and suppresses the benefits of exercise. Moderate exercise training promotes antioxidant signaling via stabilization of Nrf2, thereby protects the aging myocardium from oxidative-stress associated pathological processes.

Several studies have elaborately demonstrated that either deficiency and/or insufficient activity of Nrf2 during aging resulted in impaired cell's ability to detoxify the oxygen radicals affecting the redox homeostasis leading to OS and/or oxidant sensitivity in the myocardium and heart failure (Warabi et al., 2007; Collins et al., 2009; Ungvari et al., 2011; Valcarcel-Ares et al., 2012; Zhou et al., 2014). Further, disturbances in redox homeostasis have been shown to induce apoptosis and/or necrosis of myocyte resulting in decreased myocyte number, a hallmark of aging heart that in turn, results in remodeling and hypertrophy (Kajstura et al., 1996). Thus, a combination of two causes of increased ROS (i.e., aging and heart's intrinsic richness in mitochondria, a seat of oxidative metabolism to generate ROS) can present a high degree of complexity and challenge, and thus such a mechanism involving Nrf2 to control the oxidative burden in aging heart becomes an important issue. It is thus clear that Nrf2 dysregulation is at the intersection of both aging and cardiovascular pathology wherein, decline in Nrf2-antioxidant pathway during aging is shown to predispose the cardiac tissues to various adverse etiologies of disease development (Higami and Shimokawa, 2000; Zhou et al., 2014). Given that in the physically fit who undergoes progressive training, there is a decreased incidence of OS based pathologies due to establishment of adaptive resistance to OS along with induction of trophic factors and oxidative-damage repairing systems (Radak et al., 2001, 2009; Katzmarzyk and Janssen, 2004; Halestrap et al., 2007; Tremblay et al., 2007; van Praag, 2008), this review aims to focus on the systematic functions (up and down regulation) of Nrf2 with respect to different mode of exercises such as acute exercise stress (AES), endurance exercise stress (EES) and moderate exercise training (MET), and sketch the importance of maintaining “OPTIMAL” Nrf2 expression for the healthy regulation of redox homeostasis under disparate provocations in the cardiac system (Figure 2).

## Acute exercise stress (AES) vs. Nrf2 antioxidant signaling

Exercise is a well-known means of tissue oxygenation that is shown to elevate maximal oxygen uptake and improve the cardiac fitness (Myers, 2003). However, the gain of cardiac fitness is shown to be a strong correlate of the extent of the physical activity (Eaton, 1992). In search of the mechanisms underlying exercise-induced improvement in fitness, subsequent studies, demonstrated that ROS generated can activate different redox-sensitive transcription factors, including Nrf2, NF-κB, and reinforce the antioxidant system (van Empel et al., 2005; Berzosa et al., 2011; Muthusamy et al., 2012; Narasimhan et al., 2014). Prior research points out an impairment of the antioxidant defense mechanisms due to either an absence or declined activation of Nrf2, as a cause for poor cardiac performance, pressure overload-induced dysfunction, cardiac fibrosis and inflammation through elicited OS (Li et al., 2009; Ungvari et al., 2011; Gounder et al., 2012). In connection with this, a previous study by our group demonstrated that acute exercise training mildly induced oxidative state rather stress leading to nuclear translocation of Nrf2 and transcriptional activation of Nrf2-regulated antioxidant enzymes in the heart of WT mouse (Muthusamy et al., 2012). However, in the same study, the genetic depletion of Nrf2 failed to demonstrate the compensatory cytoprotective adaption along with high OS in response to AES when compared with WT. What is apparent from the study is the fact that exercise may exert a beneficial effect in protecting the myocardium only if the Nrf2-dependent EpRE/ARE signaling pathway is intact. Notably, this study was performed in young mice of ~2 months old. Relative to younger ones, aged individuals display several cardiac structural and functional changes and are highly susceptible to different cardiac stress. In addition, the exercise tolerance is shown to progressively decline with increasing age (Strait and Lakatta, 2012) with the peak maximal oxygen capacity (VO_2_) declines in both the less active and highly active healthy aged individuals impacting the cardio-respiratory fitness (Fleg et al., 1994, 2005; Hollenberg et al., 2006; Jackson et al., 2009). Given an age-associated decline in heart function and exercise tolerance, we followed up our earlier study and demonstrated that the basal Nrf2 and its dependent ARE signaling is reduced in aging (Gounder et al., 2012). Importantly, the aging mice could not adapt to AES training due to either insufficient Nrf2 levels and/or exhaustion of existing antioxidant pool to overcome the AES stress (Figure 2). A reduced response to acute exercise in healthy older men was already reported due to decreased β-adrenergic responses (Stratton et al., 1994). We are aware of a recent report demonstrating that β1-adrenergic receptors mediate Nrf2/HO-1/HMGB1 axis against hypoxia/reoxygenation (H/R)-induced neonatal rat cardiomyocytes injury (Wang et al., 2015). Thus, it would be interesting to evaluate in the future as to how and what level of Nrf2 signaling interacts with β-adrenergic signaling in acute exercise training during aging.

## Endurance exercise stress vs. Nrf2-antioxidant signaling in the heart

Endurance exercise stress (EES) has been suggested as an attractive strategy to mitigate the onset of sarcopenia with age (Lupien et al., 1998) and attenuate mitochondrial dysfunction in aging and associated comorbidities (Safdar et al., 2011). However, a randomized, single-blinded clinical trial demonstrated that endurance training was either “neutral” or “negative” with no significant improvement in maximal oxygen consumption (VO_2_), left ventricular (LV) structure and function. Given the inconsistent effect of endurance training besides the duration and intensity of exercise regimen exerting differential influences, we curiously conducted EES study to investigate the Nrf2 signaling dependent redox homeostasis in the aging heart (Gounder et al., 2012). The main findings of this study are that when the young-adult (6 months old) mice were stressed to their endurance capacity, a promotion of Nrf2 signaling and reinforcement of redox homeostasis in the heart was observed. In contrast, the levels of Nrf2 and its target gene expression analysis in old mice that were subjected to EES revealed significant downregulation of most of the myocardial antioxidant genes (Gounder et al., 2012). The prominent targets of Nrf2 namely Nqo1 and Ho1 along with the genes encoding the subunits of γ-glutamyl cysteine ligase (GCL), the rate-limiting enzyme for GSH biosynthesis, Gclm and Gclc were significantly repressed in the aging heart when compared to young mice following EES. The ROS scavenging enzymes such as Sod2, catalase and Gpx1 were also blunted in the old mice. Induction of mRNA levels for G6PD and GSR, key enzymes responsible for recycling oxidized glutathione (GSSG) back into its reduced form (GSH), exhibited a similar trend, being increased in young, but blunted in old mice following EES. The protein levels of these antioxidant genes correlated with their transcript levels along with exacerbated ROS levels indicating a tight regulation of Nrf2 signaling, which is impaired in the aging heart following EES. These results underscore that aged hearts that is marked by a reduction and/or insufficient Nrf2 signaling may not overcome the EES-induced oxidative capacity and thus can result in long-term changes in the structure and function of the myocardium. Although endurance training is reported to display better VO_2_ in older athletes when compared to their sedentary aged counterparts but not to that of younger ones (Fleg et al., 1994, 2005), our animal study indicates that a discrete Nrf2 gene content (either presence or absence) can have a major influence in determining the endurance training outcome. However, despite the fact that healthy older adults can relatively enjoy the benefits of exercise training, how far the same physical training can be feasible and/or advantageous in elders with a pre-existing heart condition? In connection with this, studies are underway in our laboratory to identify the interaction of energy balance, EES and aging using non-exercise based calorie restriction model to improve cardiac health since the energy expenditure associated with physical activity is widely thought to be involved in influencing the signaling changes and its beneficial effect (Figure 2).

## Moderate exercise training (MET) vs. Nrf2-antioxidant signaling

Having demonstrated the involvement of Nrf2 during short sub-maximal (acute) strategy and maximal endurance training in aging animals, we also conducted MET for an extended period of time and evaluated the role of Nrf2 signaling in the young vs. aging heart. In response to prolonged, but moderate, exercise for 6-weeks, nuclear Nrf2 protein was significantly increased in the hearts of both young and old mice, indicating that moderate training-induced stability of Nrf2. This was associated with an increase in the transcript and subsequently, the protein levels of major targets of Nrf2 (GSR, HO1, G6PD, GPX1, and GCS), suggesting that the nuclear-translocated Nrf2 is functionally active. Although the exercise-mediated induction of Nrf2-dependent antioxidant targets in aged mice was not as robust as that seen in the younger group, they were significantly increased relative to the sedentary aged group and also when compared with the EES-trained aged animals (Laughlin and McAllister, 1992; Gounder et al., 2012). Prior studies have demonstrated that a short- and long-term MET in animals and humans with ischemic heart disease, and cardiac heart failure showed improvements in myocardial perfusion and functional capacity (Laughlin and McAllister, 1992; Belardinelli et al., 1998, 1999). Notably, MET has been suggested to be beneficial in preventing cardiovascular disease in aging (Bean et al., 2004; Navarro et al., 2004); however, none elucidated the precise mechanism. Studies on MET in aging animals from our laboratory suggest that it is the improved Nrf2 signaling and the resultant restoration of redox homeostasis relatively similar to those that is observed in the younger animals could be one of the potential reasons for MET's counter effect for cardiac aging (Gounder et al., 2012). Just prior to our same observation (Gounder et al., 2012), there was an interesting finding which utilized a five times each week swimming system in warm water at 30–32°C with a duration of 60 min for 10 weeks that is categorized under MET demonstrating a functional and structural improvements of the cardiovascular system through a suggested neovascularization (including angiogenesis and vasculogenesis; Deschenes and Ogilvie, 1999; Whyte and Laughlin, 2010; Padilla et al., 2011). Now with our understanding that MET enhances Nrf2 in aging model and given a strong proangiogenic role for Nrf2 through activating HIF-1α/VEGF pathway in cancer pathobiology (Kim et al., 2011; Zhang et al., 2013), it would be interesting to investigate if and how MET can integrate Nrf2 with angiogenesis during aging to improve cardiac fitness.

## Conclusions

Based on the recent literature on exercise and cardioprotection, multiple notions have been proposed by independent investigators (Fisher-Wellman and Bloomer, 2009; Kendall and Fairman, 2014). For instance, while endurance exercise is a reasonably well-established intervention to improve the tolerance of the myocardium and vasculature against oxidative injury, recent studies show evidence for detrimental outcomes after chronic endurance exercise protocols (Pina et al., 2003; La Gerche et al., 2012). We hypothesize that such chronic endurance exercise may promote irreversible structural remodeling and lead to heart failure over time. Although abnormal levels of ROS are harmful and induce pathological cellular signaling, mild to modest concentrations that are comparable to the levels generated during acute to MET are likely to prime the transactivation of functional antioxidant adaptation.

Therefore, stress with physiological stimuli can describe the existing understanding of exercise as an example of a benefit, in which a modest grade of the pro-oxidative condition established through appropriate type of exercise could result in protective adaptation (Radak et al., 2013). Activation of Nrf2, a master transcriptional regulator of cytoprotective antioxidant defense genes is not only accountable for, but conceivably essential for, the benefits of exercise. Although, a natural link between Nrf2 and redox signaling in response to exercise is emerging, it is challenging to explain the amount of stress produced by a specific type of exercise and its consequence in relation to cellular adaptation in a given setting i.e., genetic makeup involving ethnicity, gender, age and metabolic profile comprising diabetic index, lipid composition, hypertension, and other chronic illnesses etc. Nonetheless, we have reported that active to modest to high-intensity endurance exercise cause distinct effects on the antioxidant response in the mouse myocardium (Gounder et al., 2012; Muthusamy et al., 2012; Namakkal Soorappan et al., 2012). Interestingly, these outcomes hugely vary from beneficial to detrimental effects contingent on the levels and/or stability of Nrf2, and also in an age-dependent manner. Using genetic mouse models of Nrf2 deficiency or ablation, these findings confirmed the susceptibility of the Nrf2-null mice to exercise stress (Muthusamy et al., 2012). The degree of abnormal cardiac remodeling in response to exercise stress is meaningfully higher in the Nrf2-null vs. WT mice and this maladaptive outcome is further exaggerated by aging. Orchestrated induction of Nrf2-dependent gene transcription is crucial for a living cell to sustain redox homeostasis in response to any given mode of exercise training or stress. However, very relevant is the fact that the endogenous priming of the antioxidant system as against its exogenous supplementation is increasingly considered to be responsible for the health benefits associated with exercise which stems from the claims that exogenous supplementation of antioxidants produces inconsistent and/or negative ergogenic effects (Malm et al., 1997; Clarkson and Thompson, 2000; Evans, 2000; Bloomer, 2007; Braakhuis, 2012). Future investigations at cellular, biochemical, molecular, and patho-physiological levels centering Keap1-Nrf2 signaling are necessary to understand the unique effects of different modes of exercises on myocardial defense. In specific, investigations on age-related adaptations to a particular exercise are necessary to reveal the accelerated/pathological aging in cardiac complications (Figure 2).

## Author contributions

All authors listed, have made substantial, direct, and intellectual contribution to the work, and approved it for publication.

### Conflict of interest statement

The authors declare that the research was conducted in the absence of any commercial or financial relationships that could be construed as a potential conflict of interest.
